# A Retrospective Analysis of Azvudine in Patients with COVID-19 and Pre-existing Cancer

**DOI:** 10.7150/jca.91530

**Published:** 2024-03-04

**Authors:** Fangyu Li, Keao Zheng, Xueyan Qi, Kaixia Cui, Jing Yang, Zhiying Hao

**Affiliations:** 1 School of Pharmacy, Shanxi Medical University, Taiyuan 030001, China.; 2 Department of Pharmacy, Shanxi Province Cancer Hospital/Shanxi Hospital Affiliated to Cancer Hospital, Chinese Academy of Medical Sciences/Cancer Hospital, Affiliated with Shanxi Medical University, Taiyuan, 030002, China.

**Keywords:** COVID-19, Azvudine, cancer, RT-PCR, retrospective study

## Abstract

**Objectives:** Azvudine has been recommended as a potential treatment for the recently discovered Coronavirus disease (COVID-19) in 2019. However, the effectiveness of Azvudine in individuals who have both COVID-19 and pre-existing cancer remains uncertain. Consequently, we undertook a retrospective analysis to evaluate the clinical efficacy of Azvudine therapy in hospitalized patients with COVID-19 and pre-existing cancer.

**Methods:** This is a single-center retrospective analysis of patients diagnosed with severe acute respiratory syndrome coronavirus 2 (SARS-CoV-2) infection, selected from patients admitted to a specialist oncology hospital between June 1, 2022 to June 31, 2023 with positive RT-PCR and pre-existing cancer. After exclusion and propensity score matching, patients in the test group treated with Azvudine and control patients treated with standard antiviral therapy were included. The primary outcome is the interval time from the first dose of Azvudine to the attainment of the first negative result for nucleic acid. Secondary outcomes included the rate of nucleic acid conversion, the duration of hospitalization, and the admission to the intensive care unit (ICU). Cox proportional hazards models were used to analyze the hazard ratio (HR) of event outcomes and to assess whether cancer types and Azvudine treatment will affect the course of COVID-19, specifically the time it takes for primary symptoms to alleviate.

**Results:** In this study, a total of 84 patients were included for analysis. Among them, 42 patients received Azvudine treatment after hospitalization, and the rest were treated with standard antiviral therapy. The results expressed that the time taken for the first negative nucleic acid test was significantly shorter in the Azvudine group compared to the control group [5 (IQR3-7) d vs 12 (IQR9-15) d],* p<*0.0001. This difference was statistically significant. Furthermore, a multivariate COX analysis indicated that Azvudine treatment could effectively reduce the time required for nucleic acid conversion in cancer patients (HR 1.994, 95% CI 1.064-3.736, *p*=0.031). And the type of cancer also had an impact on the course of COVID-19 in patients. (HR 3.442, 95%CI 1.214-9.756,* p*=0.020; HR 3.246, 95% CI 1.925-7.209, *p*=0.036).

**Conclusion:** Azvudine was correlated with a reduced duration for achieving nucleic acid conversion in individuals diagnosed with cancer. And different types of cancer have a certain impact on the course of COVID-19 for patients.

## Introduction

The global impact of Coronavirus Disease 2019 (COVID-19), an infectious disease caused by severe acute respiratory syndrome coronavirus 2 (SARS-CoV-2), has been profound, affecting numerous countries and regions. Originating in Wuhan, Hubei Province, China in late 2019, the virus quickly spread across the globe^1^. The manifestation of symptoms typically occurs within a period of 2 to 14 days after exposure to the source of infection, ranging from mild cold-like symptoms such as fever, cough, and sore throat, to the more severe acute respiratory distress syndrome (ARDS) and even death^2^. Due to its highly contagious nature and the diverse range of clinical presentations, COVID-19 has had significant health, social, and economic consequences on a global scale.

The virus continues to mutate, with Omicron replacing Delta as the dominant “mutant of concern”. As indicated by the most recent risk assessment conducted by the World Health Organization, the omicron EG.5 variant shows higher prevalence, growth advantage, and immune escape properties^3^. Furthermore, with the dissemination of the epidemic and the rising number of infection cases, the combination of SARS-CoV-2 in oncology patients has garnered significant societal attention. These patients have emerged as the most susceptible population to infection during the pandemic due to potential complications following surgery, radiotherapy, targeted therapy, immunotherapy, and other treatments, as well as the underlying disease itself. Factors such as compromised cardiopulmonary function and malnutrition resulting from combination therapy further contribute to their vulnerability^4^. A COVID-19 prospective cohort study published in the Lancet demonstrated that oncology patients with diminished autoimmune function were particularly susceptible to pneumonia during the epidemic, facing a heightened risk of infection compared to the general population. Moreover, once infected with SARS-CoV-2, oncology patients experienced rapid progression, severe symptomatology, and elevated mortality rates^5^.

The global spread of the epidemic has posed a significant risk to human health, necessitating the immediate availability of antiviral drugs that can effectively combat all variants. Presently, there have been notable advancements in the field of drug therapies for COVID-19, both domestically and internationally. In China, particular attention has been given to Azvudine tablets as a potential treatment for COVID-19. It is noteworthy for being the first dual-targeted anti-HIV drug with a unique combination of nucleoside auxiliary protein and reverse transcriptase inhibitor^6^. In 2022, the National Medicine Products Administration (NMPA) granted approval for Azvudine as a treatment for COVID-19, marking it as the first orally administered antiviral drug to be approved in China^7^. Recommendations for the use of Azvudine in the treatment of adult COVID-19 patients were provided by the National Health Commission (NHS) and the National Administration of Traditional Chinese Medicine (NATCM). Prior to this approval, clinical investigations into the therapeutic effectiveness of Azvudine for COVID-19 were initiated in 2020, with phase III clinical trials conducted in China, Russia, and Brazil. The results of these trials indicated that patients experienced significant reductions in viral load and notable reductions in the time required for clinical symptom remission on days 3, 5, and 7 following the administration of 5 mg/d of Azvudine^8^. However, whether Azvudine can effectively reduce the duration of COVID-19 in hospitalized cancer patients remains unknown. Consequently, we conducted a retrospective study to preliminarily explore the efficacy of Azvudine in COVID-19 cancer patients.

## Materials and methods

### Study Design and Patients

This scholarly analysis is based on a retrospective study conducted at a tertiary-level oncology specialty hospital in Shanxi Province, focusing on COVID-19 patients with cancer. The study took place from June 1, 2022 to June 31, 2023 and obtained approval from the Hospital Institutional Review Board (2023WJW23). In this retrospective cohort study, patient anonymity was ensured, and individual informed consent was not required. The study included patients who met certain criteria: 1) Those with at least one pre-existing cancer; 2) Age ≥ 18 years; 3) Hospitalized patients with a positive RT-PCR for SARS-CoV-2 infection; 4) Receiving Azvudine therapy. We excluded participants who were: 1) Age < 18 years; 2) Receiving antiviral medications other than Azvudine; 3) Those patients diagnosed as severe or critical upon admission (severe cases were defined as respiratory rate ≥30 breaths/min, or oxygen saturation ≤93%, or PaO2/FiO2 ≤ 300 mmHg, or pulmonary infiltrates >50%). 4)Patients with known or suspected allergy to the ingredients of Azvudine tablets, pregnancy, and patients with contraindications.

### Data collection

We retrieved electronic health records for COVID-19 patients from the hospital inpatient system. These records contained various information such as the patients' demographic characteristics, date of admission, previous illnesses, prescription and medication reconciliation records, and symptoms at the time of admission. Also, the patient's laboratory tests and date of discharge or death were collected. Data collection was ongoing until the planned point in time.

### Control and Matched

Controls were selected from hospitalized COVID-19 patients who did not receive Azvudine or any other antiviral medications during the observation period. Propensity score matching was used in a 1:1 ratio. The baseline covariates for patients included age, gender, types of cancer in patients (classified based on location into head and neck tumors, respiratory system tumors, digestive and genitourinary tumors, and hematologic system tumors), concomitant therapy upon admission (such as antibiotics, nutritional support, antithrombotic drugs and hematopoietic growth factors and immunomodulators).

### Outcomes

The primary outcome was the time from the first administration of the drug to the first nucleic acid negative test in COVID-19 cancer patients. Nasopharyngeal swab nucleic acid testing was conducted daily. Secondary measures of observation included the rate of nucleic acid conversion after drug therapy, the length of hospital stay, and the admission to the intensive care unit. The nucleic acid conversion rate was defined as the proportion of patients in the study with negative nucleic acid tests compared to all patients in the group.

### Statistical analyses

Data analysis was processed using Excel 2021 statistical software and statistically analyzed using SPSS.25.0 statistical software. The Shapiro-Wilk test was used to assess the normality of the distribution of continuous variables, and measures that conformed to normal distribution were expressed as mean ± standard deviation (xs), Comparison between the two groups was performed using t-test. For continuous that didn't follow a normal distribution, they were expressed as median (interquartile spacing IQR) and analyzed using the Mann-Whitney U test. Categorical variables are expressed as absolute numbers (percentages). The chi-square test (χ^2^-test) was used to compare the count data between groups. A propensity score model conditional on baseline characteristics was used to control for confounders between the two groups. Cox proportional hazards models were applied to assess the association between Azvudine treatment and nucleic acid-negative conversion, with variables including age (<60 or ≥60 years), sex, group (Azvudine group or control group), and cancer types (head and neck tumors, respiratory system tumors, digestive and genitourinary tumors, and hematologic system tumors), which were tested by multivariate analysis. The Kaplan-Meier method was used to show the time curve of the first negative nucleic acid test within 20 days after drug treatment. The log-rank test was used to compare the difference in time to the first negative nucleic acid test curve between the two groups. Graphs were plotted using Graphpad Prism 9.5 software. *p* < 0.05 was considered statistically significant.

## Results

### General information

In this study, a total of 52 patients in the Azvudine group and 55 patients in the control group were collected. To account for potential confounding factors, the propensity score matching method was employed, resulting in the successful matching of 42 pairs. Following the matching process, a comparison was made between the two groups in terms of gender, age, cancer types, and concomitant treatments upon admission. The results indicated that there were no statistically significant differences (*p* > 0.05) between the groups, and they were considered comparable, as shown in Table 1.

### Clinical outcomes

The clinical outcomes of the patients are shown in Table 2. All patients survived. The time from initiation of treatment to the first negative nucleic acid test was shorter in the patients treated with Azvudine compared with the control group [5 (IQR: 3-7) days vs. 12 (IQR: 9-15) days, *p* < 0.001]. The median length of hospitalization was 16 days (IQR: 11.75-37) in the Azvudine group and 18 days (IQR: 9.75-31.25) in the control group, which was not statistically significant (*p=*0.258). The nucleic acid conversion rate was 64.3% in the Azvudine-treated group compared with 50% in the control group. The difference between the two groups was also not statistically significant in terms of indicators of progression to severe or critical illness (*p*=0.397).

**Figure 1:**
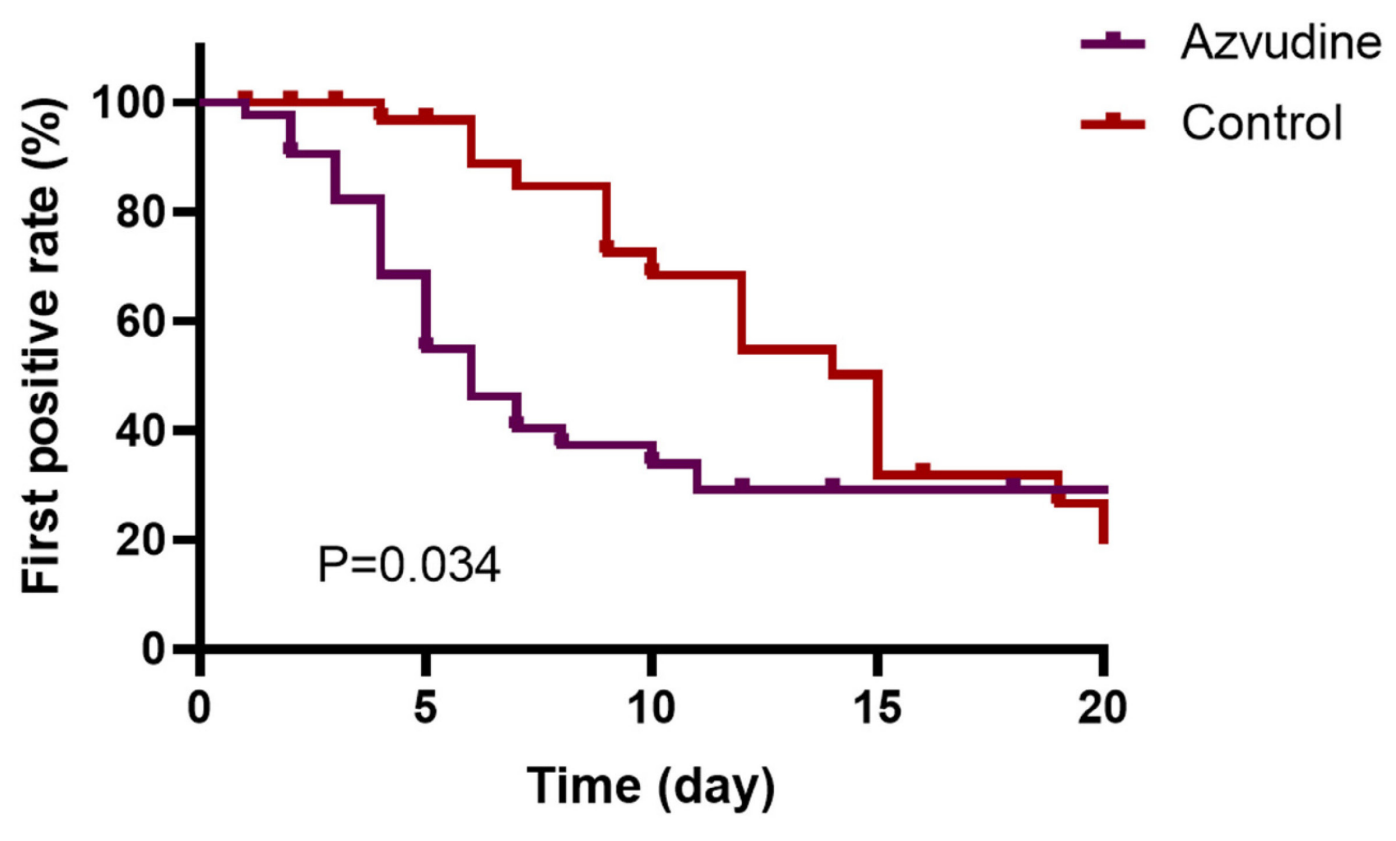
Kaplan-Meier curves of nucleic acid testing negativity within the first 20 days after drug treatment.

Multivariate Cox analysis showed that Azvudine treatment was associated with time to nucleic acid conversion during hospitalization (Table 3, HR=1.994; 95% CI: 1.064-3.736, *p=*0.031). The Kaplan-Meier survival curves illustrated that Azvudine treatment could increase the nucleic acid negative conversion rate within the first 20 days after drug treatment (Figure 1, P_log-rank test_=0.034). Additionally, COX analysis showed that digestive and genitourinary tumors, hematologic system tumors have an impact on the duration of ARS-CoV-2 disease in patients (HR=3.442; 95% CI: 1.214-9.756, *p* =0.020; HR=3.246; 95% CI: 1.925-7.209, *p* =0.036).

## Discussion

The Diagnosis and treatment protocol for COVID-19 in China (trial version 10)^9^ incorporates Nirmatrelvir/Ritonavir tablets and Azvudine, a domestically developed small molecule drug in China, into the antiviral drug regimen. Currently, Nirmatrelvir/Ritonavir remains the preferred antiviral treatment for COVID-19 infections^10^. However, due to its high cost and limited availability, an increasing number of patients are being treated with Azvudine. Multiple clinical trials have provided evidence that Azvudine effectively inhibits SARS-CoV-2 replication and enhances immune function in COVID-19 patients. This leads to a shorter duration for nucleic acid-negative conversion in mild and common COVID-19 patients^11^, ^12^. This scholarly study conducted at a single center retrospectively assesses the effectiveness of Azvudine tablets in patients with COVID-19 and cancer. The current body of research on the correlation between Azvudine and COVID-19 in cancer patients is limited, making this study the first real-world investigation of Azvudine's impact on individuals with SARS-CoV-2 and concomitant tumors. Our findings indicate that there was no significant disparity in hospitalization duration between the group receiving Azvudine and the control group [16 (IQR: 11.75-37)d vs 18 (IQR: 9.75-31.25)d, *p=*0.258]. But the generally longer length of hospitalization is in line with the longer hospitalization course of cancer patients as elucidated in previous studies^13^. Prolonged hospitalization in cancer patients relative to non-cancer patients patients may be due to the heightened presence of additional medical conditions. COVID-19 is essentially a self-limiting viral infection and gradually regresses over time, with the largest proportion of mildly ill patients^14^, which was reflected in our study, with a significant proportion of COVID-19 patients experiencing mild symptoms, which was consistent in both groups of patients examined. Meanwhile, from our research, it was found that for tumor patients infected with COVID-19, the time from first drug use to first RT-PCR negative conversion is often shorter for patients receiving Azvudine treatment compared to those receiving standard treatment. In the COX proportional hazards model, we analyzed basic information like age, gender, cancer types, and medication status (Azvudine group or control group). Among these variables, Azvudine is an influencing factor, and the Kaplan-Meier curve showed a difference in survival distribution between the Azvudine group and the control group, meaning that Azvudine treatment can shorten the nucleic acid conversion time for COVID-19 cancer patients. This finding is similar to the research results of Sun et al^15^, whose retrospective study results showed that Azvudine has a protective effect on COVID-19 and patients with previous diseases, and Azvudine treatment can significantly reduce the risk of disease progression outcomes. Furthermore, our Cox analyses revealed a correlation between digestive and genitourinary tumors, hematologic system tumors, and the duration of patients' COVID-19 disease. This implies that different types of cancer can also affect the duration of patients' disease, which aligns with previous research that has shown the impact of R/R B-cell lymphomas on the survival of COVID-19 patients 16. Importantly, it should be noted that there was no observed rise in the occurrence of severe COVID-19 pneumonia among a group of patients receiving cancer treatment for COVID-19. The study is, nonetheless, constrained by a limited sample size, particularly the bias in sample selection resulting from this small size. The batch of COVID-19 cancer patients we collected represents the patient population at the Shanxi Province Cancer Hospital. A nationwide study involving multiple centers and a larger patient cohort may provide a more representative population, which could further determine the correlation between Azvudine treatment and COVID-19 patients combined with cancer. Furthermore, there were no differences in the demographic and baseline characteristics between the two groups of patients. However, due to missing data, we were unable to analyze the impact of some other factors on disease progression in patients, such as smoking, alcohol consumption, and obesity. Regression analysis revealed that COVID-19 cancer patients who were obese and actively smoked had an increased risk of mortality in the cohort^13^. Additionally, no significant adverse reactions related to Azvudine were observed.

## Conclusion

In conclusion, the findings of this single-center study show that Azvudine can shorten the duration of nucleic acid conversion in cancer patients. It is clinically relatively safe and brings hope to COVID-19 cancer patients. Also, the combination of different cancer types had an impact on the course of COVID-19 patients. We hope that there will be larger-scale studies in the future to support our viewpoint.

## Figures and Tables

**Table 1:** Baseline characteristics of both groups of participants.

Baseline characteristics	Before matching			After propensity score matching		
	Azvudine (n=52)	P	Controls (n=55)	Azvudine (n=42)	P	Controls (n=42)
Age (years), mean (SD)	63.38±10.552	0.003	57.16±12.546	61.93±10.84	0.208	59.71±12.19
Gender, n (%)		0.015			0.620	
Female	21 (40.4)		13 (23.6)	15 (35.7)		12 (28.6)
Male	31 (59.6)		42 (76.4)	27 (64.3)		30 (71.4)
Cancer types, n (%)						
Head and neck tumors	7 (13.5)	0.072	2 (3.6)	3 (7.1)	0.645	2 (4.7)
Respiratory system tumors	14 (26.9)	0.259	23 (41.8)	13 (31.0)	0.118	20 (47.6)
Digestive and genitourinary tumors	22 (42.3)	0.459	21 (38.2)	17 (40.4)	0.498	14 (33.3)
Hematologic system tumors	9 (17.3)	0.933	9 (16.4)	9 (21.5)	0.393	6 (14.4)
Concomitant therapy, n (%)						
Nutritional support	16 (30.8)	0.232	11 (20.0)	12 (28.6)	0.620	10 (23.8)
Antibiotics	7 (13.5)	0.503	11 (20.0)	6 (14.4)	0.266	10 (23.8)
Antithrombotic	10 (19.2)	0.383	7 (12.7)	7 (16.5)	0.763	6 (14.3)
Hematopoietic	4 (7.7)	0.546	6 (10.9)	4 (9.5)	0.724	5 (12.0)
Immunomodulators	15 (28.8)	0.372	20 (36.4)	13 (31.0)	0.629	11 (26.1)

**Table 2:** Clinical outcomes of study patients

	Controls (n=42)	Azvudine (n=42)	P(t/χ^2^) values
Proportion of nucleic acid negative conversion, n (%)	20 (47.6)	27 (64.3)	0.124
Intensive care unit admission, n (%)	4 (9.5)	2 (4.8)	0.397
Time from drugs treatment to first nucleic acid negative conversion	12 (9-15)	5 (3-7)	<0.0001
Length of hospital stay	18 (9.75-31.25)	16 (11.75-37)	0.258

**Table 3:** Cox proportional hazards model variables for the rate of nucleic acid negative conversion during hospitalization

	Number (%)	Multivariable analysis	
		Hazard ratio (95%CI)	P value
**Group**			
Control group	42 (50)	Reference	
Azvudine group	42 (50)	1.994 (1.064-3.736)	0.031
**Age**			
≥60	51 (60.7)	Reference	
<60	33 (39.3)	0.770 (0.407-1.456)	0.421
**Gender**			
Male	57 (67.9)	Reference	
Female	27 (32.1)	1.999 (0.990-4.037)	0.053
**Cancer types, n(%)**			
Head and neck tumors	4 (4.8)	3.565 (0.778-6.334)	0.102
Respiratory system tumors	29 (34.5)	2.539 (0.793-8.127)	0.117
Digestive and genitourinary tumors	36 (42.9)	3.442 (1.214-9.756)	0.020
Hematologic system tumors	15 (17.8)	3.246 (1.925-7.209)	0.036
